# Supplementary material for: The spatial heterogeneity of urban green space distribution and configuration in Lilongwe City, Malawi
Source: PLoS One. 2024 Jul 24;19(7):e0307518. doi: 10.1371/journal.pone.0307518 (PMC11268670; doi:10.1371/journal.pone.0307518)
Supplement: Attachment — Submitted filename: PONE-D-23-36331_reviewer_1.pdf [file pone.0307518.s001.pdf]

PLOS ONE

The spatial heterogeneity of urban green space distribution and configuration in Lilongwe City, Malawi

--Manuscript Draft--

**Manuscript Number:**	PONE-D-23-36331
**Article Type:**	Research Article
**Full Title:**	The spatial heterogeneity of urban green space distribution and configuration in Lilongwe City, Malawi
**Short Title:**	The spatial heterogeneity of urban green space distribution and configuration
**Corresponding Author:**	Odala Nambazo, MSc.<br>Ministry of Agriculture and Food Security<br>Lilongwe, Lilongwe MALAWI
**Keywords:**	Urban green spaces; configuration; neighbourhoods; functional land uses; disparities; urban form
**Abstract:**	Urban green spaces provide several benefits related to the quality of urban life. The existence and spatial arrangement of these spaces within neighbourhoods and functional land uses have significant implications for the well-being of urban dwellers. Previous studies on green spaces in urban areas of Malawi have focused on a broader and a macro-level perspective offering insightful information on general trends in different cities. However, there is a significant research shortage in localised understanding, which requires carrying out micro-level assessments concentrating on land use zones and neighbourhoods within these cities. In this study, we used remote sensing data and landscape metrics to unravel the distribution of urban green spaces in the city and understand the distribution and configuration of urban green spaces in the city's neighbourhoods and functional land uses and their relationship with urban form. The study results revealed that 20% of neighbourhoods fail to meet the WHO-recommended standard of 9 m2 of green space per person, with a predominant concentration of these undersupplied areas in high-density and quasi-residential zones. In addition, 56.2% of Lilongwe City's total green area was contained under functional land uses. Particularly, high-rise residential, medium-density residential, low-density residential, quasi-residential, high-rise flat area, commercial class, high-rise commercial, heavy industry, light industry, and government land use zones contained 17.3%, 12.0%, 22.2%, 12.0%, 4.1%, 6.4%, 6.1%, 5.0%, 1.6%, and 13.3% of the total green spaces in functional land uses, respectively. Importantly, this research unveils the significant correlation of urban form metrics, namely building coverage, building density, building perimeter area ratio, road density, and the distribution and configuration of urban green spaces. This necessitates an integrated approach to urban planning and design, emphasizing the importance of balancing development with green space preservation.
**Order of Authors:**	Odala Nambazo, MSc.
	Kennedy Nazombe, MSc
**Additional Information:**	
**Question**	**Response**
**Financial Disclosure**<br><br>Enter a financial disclosure statement that describes the sources of funding for the work included in this submission. Review the submission guidelines for detailed requirements. View published research articles from *PLOS ONE* for specific examples.	The author(s) received no specific funding for this work.

Powered by Editorial Manager® and ProduXion Manager® from Aries Systems Corporation

This statement is required for submission and **will appear in the published article** if the submission is accepted. Please make sure it is accurate.<br><br>**Funded studies**<br>Enter a statement with the following details:<br>• Initials of the authors who received each award<br>• Grant numbers awarded to each author<br>• The full name of each funder<br>• URL of each funder website<br>• Did the sponsors or funders play any role in the study design, data collection and analysis, decision to publish, or preparation of the manuscript?<br><br>Did you receive funding for this work?	
**Competing Interests**<br><br>Use the instructions below to enter a competing interest statement for this submission. On behalf of all authors, disclose any competing interests that could be perceived to bias this work—acknowledging all financial support and any other relevant financial or non-financial competing interests.<br><br>This statement is required for submission and **will appear in the published article** if the submission is accepted. Please make sure it is accurate and that any funding sources listed in your Funding Information later in the submission form are also declared in your Financial Disclosure statement.<br><br>View published research articles from *PLOS ONE* for specific examples.	The authors have declared that no competing interests exist.

**NO authors have competing interests**<br><br>Enter: *The authors have declared that no competing interests exist.*<br><br>**Authors with competing interests**<br><br>Enter competing interest details beginning with this statement:<br><br>*I have read the journal's policy and the authors of this manuscript have the following competing interests: [insert competing interests here]*<br><br>* typeset	
**Ethics Statement**<br><br>Enter an ethics statement for this submission. This statement is required if the study involved:<br><br>• Human participants<br>• Human specimens or tissue<br>• Vertebrate animals or cephalopods<br>• Vertebrate embryos or tissues<br>• Field research<br><br>Write "N/A" if the submission does not require an ethics statement.<br><br>General guidance is provided below. Consult the submission guidelines for detailed instructions. **Make sure that all information entered here is included in the Methods section of the manuscript.**	N/A

**Format for specific study types**<br><br>**Human Subject Research (involving human participants and/or tissue)**<br>• Give the name of the institutional review board or ethics committee that approved the study<br>• Include the approval number and/or a statement indicating approval of this research<br>• Indicate the form of consent obtained (written/oral) or the reason that consent was not obtained (e.g. the data were analyzed anonymously)<br><br>**Animal Research (involving vertebrate animals, embryos or tissues)**<br>• Provide the name of the Institutional Animal Care and Use Committee (IACUC) or other relevant ethics board that reviewed the study protocol, and indicate whether they approved this research or granted a formal waiver of ethical approval<br>• Include an approval number if one was obtained<br>• If the study involved *non-human primates*, add *additional details* about animal welfare and steps taken to ameliorate suffering<br>• If anesthesia, euthanasia, or any kind of animal sacrifice is part of the study, include briefly which substances and/or methods were applied<br><br>**Field Research**<br><br>Include the following details if this study involves the collection of plant, animal, or other materials from a natural setting:<br>• Field permit number<br>• Name of the institution or relevant body that granted permission	
**Data Availability**<br><br>Authors are required to make all data underlying the findings described fully available, without restriction, and from the time of publication. PLOS allows rare exceptions to address legal and ethical concerns. See the PLOS Data Policy and FAQ for detailed information.	Yes - all data are fully available without restriction

Powered by Editorial Manager® and ProduXion Manager® from Aries Systems Corporation

A Data Availability Statement describing where the data can be found is required at submission. Your answers to this question constitute the Data Availability Statement and **will be published in the article**, if accepted.<br><br>**Important:** Stating 'data available on request from the author' is not sufficient. If your data are only available upon request, select 'No' for the first question and explain your exceptional situation in the text box.<br><br>Do the authors confirm that all data underlying the findings described in their manuscript are fully available without restriction?	
**Describe where the data may be found in full sentences. If you are copying our sample text, replace any instances of XXX with the appropriate details.**<br><br>• If the data are **held or will be held in a public repository**, include URLs, accession numbers or DOIs. If this information will only be available after acceptance, indicate this by ticking the box below. For example: *All XXX files are available from the XXX database (accession number(s) XXX, XXX.).*<br>• If the data are all contained **within the manuscript and/or Supporting Information files**, enter the following: *All relevant data are within the manuscript and its Supporting Information files.*<br>• If neither of these applies but you are able to provide **details of access elsewhere**, with or without limitations, please do so. For example:<br><br>*Data cannot be shared publicly because of [XXX]. Data are available from the XXX Institutional Data Access / Ethics Committee (contact via XXX) for researchers who meet the criteria for access to confidential data.*<br><br>*The data underlying the results presented in the study are available from (include the name of the third party*	Information will only be available after acceptance

Powered by Editorial Manager® and ProduXion Manager® from Aries Systems Corporation

and contact information or URL).<br>• This text is appropriate if the data are owned by a third party and authors do not have permission to share the data.<br><br>* typeset	
Additional data availability information:	Tick here if your circumstances are not covered by the questions above and you need the journal’s help to make your data available.

Powered by Editorial Manager® and ProduXion Manager® from Aries Systems Corporation

Manuscript

Click here to access/download;Manuscript;Manuscript_Odala.docx

The spatial heterogeneity of urban green space distribution and configuration in Lilongwe City, Malawi

Odala Nambazo[1*] and Kennedy Nazombe[2]

1. Department of Land Resources Conservation, Ministry of Agriculture, Lilongwe, Malawi
2. Faculty of Life Sciences and Natural Resources, Department of Land and Water Resources, Natural Resources College, Lilongwe University of Agriculture and Natural Resources, Lilongwe, Malawi

[*]Corresponding author

Email: nambazo@yahoo.com (ON)

// Abstract

Urban green spaces provide several benefits related to the quality of urban life. The existence and spatial arrangement of these spaces within neighbourhoods and functional land uses have significant implications for the well-being of urban dwellers. Previous studies on green spaces in urban areas of Malawi have focused on a broader and a macro-level perspective offering insightful information on general trends in different cities. However, there is a significant research shortage in localised understanding, which requires carrying out micro-level assessments concentrating on land use zones and neighbourhoods within these cities. In this study, we used remote sensing data and landscape metrics to unravel the distribution of urban green spaces in the city and understand the distribution and configuration of urban green spaces in the city's neighbourhoods and functional land uses and their relationship with urban form. The study results revealed that 20% of neighbourhoods fail to meet the WHO-recommended standard of 9 m^2 of green space per person, with a predominant concentration of these undersupplied areas in high-density and quasi-residential zones. In addition, 56.2% of Lilongwe City's total green area was contained under functional land uses. Particularly, high-rise residential, medium-density residential, low-density residential, quasi-residential, high-rise flat area, commercial class, high-rise commercial, heavy industry, light industry, and government land use zones contained 17.3%, 12.0%, 22.2%, 12.0%, 4.1%, 6.4%, 6.1%, 5.0%, 1.6%, and 13.3% of the total green spaces in functional land uses, respectively. Importantly, this research unveils the significant correlation of urban form metrics, namely building coverage, building density, building perimeter area ratio, road density, and the distribution and configuration of urban green spaces. This necessitates an integrated approach to urban planning and design, emphasizing the importance of balancing development with green space preservation.

Keywords: Urban green spaces; configuration; neighbourhoods; functional land uses; disparities; urban form

Introduction

Urbanisation is the key characteristic of the twenty-first century, as people migrate to cities worldwide in search of better living conditions and employment opportunities [1]. However, there are several consequences of rapid urbanisation, including increased environmental degradation, increased socioeconomic disparity, and the loss of natural areas that are essential to the well-being

of urban residents [1,2]. As cities expand and change, the design and distribution of urban green areas become more key factors that impact the quality of life within them [3,4].

Urban green space equity is becoming increasingly crucial to the sustainable development of cities as they grow. Urban green spaces can be anything from parks and gardens to greenways. Urban green spaces offer multiple benefits to city dwellers, such as improved air quality [5], recreational opportunities [6], ecological balance [7], aesthetic appeal [8], educational opportunities [9], and enhanced psychological well-being [10]. To maintain harmonious coexistence between built and natural environments, urban planners and designers nowadays are interested in considering not only the functional requirements of urban green areas but also aesthetic and ecological factors.

Numerous studies have linked the variability of urban green spaces across different land use zones and neighbourhoods to several factors. For instance, urbanisation, population density, and land value play pivotal roles in the availability of urban green space in various locations [11–13]. In addition, the distribution of green areas within different zones is affected by environmental, governance, regulatory policies, and zoning restrictions [14–16]. Socioeconomic factors, including race and income disparities, can also influence the availability and quality of green spaces [17–20]. Since the distribution of green space in urban areas tends to be uneven due to numerous factors, mapping the current state of green space is necessary for urban planners to create sustainable cities.

The previous research on green space and land use/land cover in Malawian cities and towns has mostly provided a macro-level perspective [21–24], offering insightful information on general trends in different cities. However, there is a significant research shortage in localised understanding, which requires carrying out micro-level assessments concentrating on land use zones or neighbourhoods within these cities. Such a localised approach is essential because it recognises that the dynamics of green spaces can differ significantly within a city's boundaries, depending on a variety of variables such as historical development, changing land use regulations, community-driven initiatives, and social-economic factors [11,16,17,19,25]. This knowledge gap underscores the need for studies that delve deeper into the specific settings of neighbourhoods or communities, providing a deeper comprehension of how green spaces are distributed in different areas within the cities.

Therefore, the current study assesses the distribution and composition of urban green spaces in the city's neighbourhoods and functional land uses in Lilongwe, Malawi. According to the city's urban structure plan, the functional land uses included in the study were commercial, industrial, government, and residential areas. The residential areas were further classified into high-density, medium-density, low-density, quasi-residential, and high-rise residential areas. The specific objectives of the study were to analyse (1) the distribution of urban green spaces in the city's neighbourhoods and functional land uses; (2) the configuration of urban green spaces in functional land uses; and (3) the relationship between urban form and green space distribution and configuration. A thorough methodology that incorporates information from spatial analytical techniques, remote sensing images, and geographic information systems (GIS) was adopted. The findings of this study may have an impact on urban planning strategies and policies, as well as aid in the creation of more efficient plans for the distribution and management of green spaces. The study also aims to broaden our knowledge of the spatial variability in urban green space and how it impacts the design of sustainable urban environments.

Materials and methods

Study area

Lilongwe City was declared the capital of Malawi in 1975, replacing Zomba City. The city lies between 13°45′S and 14°3′ S, 33°41′E and 33°53′ E in the central region of Malawi (Fig 1). It is the largest city in Malawi with a population of 989,318 [26] and covers about 727.8 km². The city was designed based on the garden city concept and has abundant greeneries within the central part of the city [27]. The Japan International Cooperation Agency (JICA) in conjunction with the Malawi Government prepared the Urban Development Master Plan in 2010 to guide the city's development. According to the city's plan, about 22,000 ha of land was zoned for nature sanctuary, parks and recreation, greeneries, agriculture, and forestry land uses. The city is abounding in natural beauty and environmental resources, including rivers, streams, flowers, and natural trees. However, the city is experiencing rapid urbanisation compared to other cities in Malawi, which is estimated at 4% annually [28]. Just like many other cities, Lilongwe is experiencing many challenges including those caused by rapid urbanisation. Environmental deterioration, pollution, deforestation, uncontrolled development on ecologically sensitive and weak regulatory frameworks are among other challenges facing the city.

Fig 1 Location and areas of the Lilongwe city (Malawi) as well as the green spaces within it.

Data

In this study, we used a 10-metre-resolution Sentinel Level-2A imagery acquired on September 5, 2022 (Product ID: L2A_T36LXK_A028718_20220905T080208). A satellite image covering the entire Lilongwe city was obtained from the Copernicus Open Access Hub (https://scihub.copernicus.eu/). To minimise haze and acquire cloud-free satellite images of the study area, the image was acquired during the dry season. The image was then projected onto the 1984 World Geodetic System, the Universal Transverse Mercator (UTM) Zone 36S. The Sentinel Level-2A products are already subjected to atmospheric and geometric corrections. The acquired satellite images were clipped to extract study areas using the Lilongwe city boundary shapefile layer obtained from the Lilongwe City Council. We used Microsoft building footprint data, which was downloaded from https://www.microsoft.com/en-us/maps/building-footprints. The city's neighbourhood's boundary shapefile was obtained from the National Statistical Office (NSO), which was based on census tract data. There is no universally agreed definition of a neighbourhood and most studies have used census boundaries [29]. Population census data for the neighbourhoods was extracted from the 2018 Malawi Population and Housing Census report [26]. In this study, we adapted the four main functional land use categories from the city's master plan.

Table 1: Descriptions of the functional land use categories used in the study

**Functional land use categories**	**Description**
Residential (High-Density Residential, Medium Density Residential, Low-Density Residential, Quasi Residential, and High-Rise Flat Area)	Land primarily dedicated to housing, such as single-family homes, apartment complexes, and residential neighbourhoods.
Commercial	Encompasses areas where businesses and retail activities take place, such as small shops, shopping malls, restaurants, banks, and office buildings.
Industrial (Heavy/Large Scale Industry and Light Industry)	Areas where manufacturing, warehousing, and other industrial activities occur.
Government	Government land use includes government office buildings where various government functions and public services take place and includes state residences.

Methods

Normalized Difference Vegetation Index

We used the normalised difference vegetation index (NDVI) to identify green spaces in the study area. NDVI is one of the most used indicators of the presence of vegetation [20,30–33]. The NDVI index has a range of -1 to +1. The negative NDVI index indicates non-green areas, such as deserts, water, rivers, and built-up areas, whereas the positive value denotes green areas [34] an increased NDVI index value suggests more vegetation on the ground. The NDVI value can also be used to assess the condition of plants and vegetation [35]. This is because healthy plants will have higher NDVI values than stressed plants. Equation (1) was used to calculate the NDVI:

$$\text{NDVI}=\frac{\text{NIR[Band 8]-RED[Band 4]}}{\text{NIR[Band 8]+RED[Band 4]}} \tag{1}$$

Where,

NIR [Band 8] and RED [Band 4] denote reflectance in the near-infrared and red bands, respectively, of the Sentinel-2A imaging product.

Per Capita Green Space (PCGS) and Urban Green Space Index (UGSI)

To understand green space allocation for every inhabitant of a neighbourhood, we calculated per capita green space (PCGS). PCGS is widely used to assess the quality of urban environments and their impact on residents' well-being [11,24,33,36,37]. Equation (2) was used to calculate PCGS:

$$PCGS_i=\frac{G_i}{PN_i} \tag{2}$$

Where $PCGS_i$ denotes per capita green space in neighbourhood *i*, G_i denotes total green space coverage in neighbourhood *i* and PN_i denote the total population of neighbourhood *i*. We used population census data for neighbourhoods for the year 2018 [26].

Furthermore, an Urban Green Space Index (UGSI) was adopted to measure the quantity of Urban Green Spaces (UGS) in each neighbourhood. The index denotes the availability of UGS as a percentage and provides a standardised way of comparing UGS in different neighbourhoods [33]. The UGSI is computed as follows:

UGSI for i^{th} neighbourhood can be expressed as in equation (3):

$$UGSI_i = \frac{G_i}{A_i} \quad (3)$$

Where, G_i = UGS in the neighbourhood *i*, A_i = area of the ith neighbourhood (where *i* = 1 to n).

Whereas total UGS (expressed as a percentage) in a particular neighbourhood is calculated as in equation (4):

$$UGSI_T = \frac{\sum_t^n G_i}{\sum_t^n A_i} \times 100 \quad (4)$$

Urban green space landscape metrics

Guided by previous studies [38–40], the composition and configuration of urban green spaces were assessed using the following seven landscape metrics: number of patches, largest patch index (LPI), mean Euclidean nearest neighbour distance (ENN_MEAN), mean shape index (SHAPE_MN), number of patches (NP), and patch density (PD). We used FRAGSTATS 4.2 software to calculate the selected metrics of green spaces at the class level. A detailed description of the metrics is provided in Table 2.

Table 2: A detailed description of the landscape metrics

Category	Landscape Indices	Description
	Number of Patches (NP)	Quantifies the number of green patches existing in a particular study unit. The higher NP suggests more fragmentation.
	Largest Patch Index (LPI)	The proportion of the largest green patch with a study unit. It indicates the level of dominance and concentration in a study unit.
Configuration	Mean Euclidean nearest neighbour distance (ENN_MEAN)	It measures the average distance from one green space patch to the nearest green space patch. It indicates the level of dispersion or clustering of green spaces.
	Mean shape Index (SHAPE_MN)	The average shape complexity of green space patches in a study unit. More irregular shapes are associated with higher shape index values.
	Patch Density (PD)	It quantifies the total number of green space patches distributed across an area. It shows the extent of spatial distribution, connectivity, and fragmentation.

Urban form metrics

Several studies have shown significant impacts of urban form on the spatial distribution and configuration of UGS. The relationship between urban form and green spaces has implications for

the overall sustainability, liveability, and well-being of a city. Weighted density, density gradient slope, density gradient intercept, compactness, and street connectivity urban form metrics were used to understand the impact of urban form on green space accessibility in 462 metropolitan areas globally [41]. To understand the association between urban morphology and green spaces, building coverage ratio, building perimeter, number of buildings, road coverage ratio, road intersections, and road length ratio metrics were employed [42]. Similarly, address density, building density, and household density as urban form indicators were correlated with biodiversity potential and ecosystem performance indicators in five UK cities [43]. The perimeter-area ratio (PARA), road density (RD), and compound terrain complexity index were used to evaluate the impact of urban form on UGS structure [44]. In Sheffield, road length, building density, and building areas, among other factors, as predictors of the extent and quality of green spaces [30]. Therefore, four urban form metrics were employed in the study, namely: building coverage (BC), road network density (RD), mean perimeter area ratio (PARA_MN) of buildings and building density (BD). The calculation of the urban form metrics was done in ArcGIS 10.6 software.

Urban planners and designers use building coverage (BC) as one of the indicators to quantify the total area of land occupied by buildings. The amount of land covered by buildings gives information about how intensively an area is developed and how much of the land area is undeveloped. Lower building coverage enhances the preservation of open spaces, parks, and natural areas. Using Microsoft building footprint data, BC was calculated using the equation (5):

$$BC = \frac{BA}{TA} \tag{5}$$

Where BC, BA, and TA are building coverage, the total area covered by all buildings and the total area occupied by a particular functional land use category, respectively.

The road network density (RD), which is the ratio of an area's total road network's length to its land area, was determined using freely available road network data accessed from the OpenStreetMap (OSM) website (www.openstreetmap.org). The RD is calculated as in the equation (6).

$$RD = \frac{L}{A} \tag{6}$$

Where RD is the road density, L is the total length of roads in particular land use, and A is the total land area of a particular land use.

The mean perimeter-area ratio (PARA_MN) of buildings is one of the urban form indicators used to understand the efficiency and compactness of urban development. A lower PARA_MN value indicates the compactness of buildings in an area. Compact urban forms are frequently linked to effective land use, lower infrastructure costs, and encourage walkability. PARA for each building is calculated using equation (7):

$$PARA_{Building} = \frac{P_{building}}{A} \qquad (7)$$

Where PARA is the perimeter-area ratio of each building, $P_{building}$ is the perimeter of each building, and $A_{building}$ is the total area covered by the building. The mean perimeter-area ratio (PARA_MN) of buildings within a particular functional land use area was computed using equation (8):

$$PARA\_MN_{Buildings} = \frac{\sum_{i=1}^{n} PARA_{Building_i}}{n} \qquad (8)$$

Where n is the total number of buildings in a particular functional land use category.

In addition, building density (BD), which is used to measure the concentration of buildings in a particular area was adopted as one of the indicators of urban form. The BD has an impact on the distribution and composition of urban greeneries. Land for green spaces may be scarce in high-density areas, while low-density areas may provide spaces for greeneries. However, low-density environments may encourage urban sprawl. BD is calculated using equation (9) below. It is then expressed as the number of buildings per given area:

$$BD = \frac{NB}{A} \qquad (9)$$

Where BD is the building density, NB is the number of buildings in each area, and A is the total land area.

Statistical Analysis

To find an association between urban green space metrics and urban form metrics, bivariate Pearson correlation analysis was performed in IBM SPSS 20.0, and the relevant tables were prepared. The bivariate Pearson correlation indicates the statistical significance, degree, and

direction (increasing or decreasing) of a linear relationship between two continuous variables. A correlation coefficient of 1 indicates a positive correlation between the variables; a coefficient of -1 indicates a negative correlation; and a correlation coefficient of 0 indicates no association [45].

Results

Urban green space per capita distribution in the neighbourhoods in Lilongwe city

The analysis of the UGS per capita distribution showed significant variations in the availability of green space in the Lilongwe City neighbourhoods (Table 3). The study found that 20% of the neighbourhoods have UGS which is less than the World Health Organisation (WHO) requirement of 9 m^2 per person. The majority of the neighbourhoods (54.5%) are provided with over 100 m^2 of green space per inhabitant. The remaining 25.5% of neighbourhoods have between 9 and 100 m^2 of green space per person, falling between these two extremes.

Table 3: Urban green space per capita distribution in the neighbourhoods/areas in Lilongwe City (Note: Two areas were not included due to the lack of population data)

**Per Capita UGS (person/** m^2**)**	**Area Numbers (arranged in ascending order based on the PCGS)**	**No of Areas**	**% of Areas**	**Remarks**
Less than 9	57, 33, 50, 28, 36, 8, 24, 7, 23, 21 and 22	11	20.0	9 m^2/person is the WHO standard
9 - 20	56, 51, 1 and 25	4	7.3	
21 - 40	48, 49 and 38	3	5.5	
41 - 60	18, 31 and 39	3	5.5	
61 - 100	53, 29, 39 and 44	4	7.3	
Above 100	2, 4, 5, 6, 46, 54, 58, 27, 26, 47, 55, 30, 35, 41, 17, 15, 45, 9, 43, 52, 3, 37, 10, 20, 12, 34, 11, 13, 14, and 32	30	54.5	
Totals		55	100.0	

Urban green space index (UGSI) of different areas/neighbourhoods in Lilongwe City

A further analysis of the distribution of UGS in the neighbourhoods indicated different percentages of coverage (Table 4 and Fig 2). The results show that 43.9% of neighbourhoods have less than 10% of their area covered by green space, 33.3% have between 10% and 20%, 14.0% have between 21 and 30%, and 8.8% have between 30% and 33%.

Table 4: Distribution of UGSI in Lilongwe city at area/neighbourhood level

Urban Green Spaces	Area Numbers (arranged in ascending order based on the percentage of green space)	No of Areas	% of Areas
Less than 10%	57,54, 50, 28, 8, 24, 38, 27, 1, 23, 7, 21, 36, 55, 49, 48, 22, 25, 45, 26, 2, 4, 29, 39, 33	25	43.9
10-20%	56, 46, 19, 51, 16, 39, 5, 6, 35, 41, 17, 52,53, 18,58,30, 43, 44, 47	19	33.3
21-30%	9, 31, 3, 37, 12, 34, 11 and 13	8	14.0
31-43%	15, 14, 32, 10 and 20	5	8.8
Totals		57	100.0

Fig 2 Urban Green Space Distribution: (a) Urban Green Space at area level (b) Urban Green Space Index expressed in percentage at area level

Urban green space distribution and population density

We used Pearson's correlation analysis to unravel the relationship between the distribution of UGS among the city's various neighbourhoods and population density. The study's findings revealed a weak negative association between urban green space and population density (coefficient of -0.419). Areas with high population densities had low PCGS, as also seen in Fig 3. Additionally, there was a strong positive association between the Urban Green Space Index (UGSI) and per capita UGS of 0.726. Population density and urban green space per capita had a negative correlation (correlation = -0.367). This means that an increase in population density results in a reduction in urban green spaces.

Fig 3 Relationship between PCGS and population density: (a) Population density map of Lilongwe City according to Malawi population and housing census [26], (b) Per Capita Green Space distribution of Lilongwe city

Distribution of urban green space in functional land uses in Lilongwe city

The study has shown variations in the distribution of urban green spaces in functional land uses in Lilongwe City (Table 5). The study revealed that 59.7% of Lilongwe City's total green area was

contained under functional land uses. In contrast to other functional land uses, the largest proportion of green spaces are found in residential neighbourhoods. In addition, 67.6% of UGS were found in residential zones. Particularly, 17.3%, 12.0%, 22.2%, 12.0%, and 4.1% of UGS were found in high-rise flat areas, medium-density residential areas, low-density residential areas, quasi-residential areas, and low-density residential zones, respectively. Similarly, green spaces covered 12.5%, 6.6%, and 13.3% of the area in commercial, industrial, and government land uses, respectively.

Table 5: Distribution of urban green spaces in functional land uses in Lilongwe City

	Functional Land Use Types	Land Area (ha)	Land (%)	UGS Area (ha)	UGS Area (%)
1	**Residential Class**				
	1.1 High Density Residential	6608.27	28.0	490.99	17.3
	1.2 Medium Density Residential	1892.32	8.0	340.01	12.0
	1.3 Low Density Residential	2651.61	11.2	627.66	22.2
	1.4 Quasi Residential	6026.44	25.5	339.51	12.0
	1.5 High Rise Flat Area	1145.82	4.9	117.36	4.1
2	**Commercial Class**				
	2.1 Commercial Area	1600.44	6.8	180.48	6.4
	2.2 High Rise Commercial	214.58	0.9	173.84	6.1
3	**Industrial Class**				
	3.1 Heavy/Large Scale Industry	1324.83	5.6	141.45	5.0
	3.2 Light Industry	427.00	1.8	45.07	1.6
4	**Government Class**	1733.35	7.3	375.41	13.3
Totals		23624.66	100.0	2831.78	100.0

Landscape metrics analysis for the UGS for four land use classes

Spatial patterns, compositions, and configurations varied in the nine land use classes according to the landscape metric analysis results (Table 6). The number of green space patches (NP) found within each functional land use varied significantly, with the highest number of patches being recorded in the residential land use zones (10,226 patches). This indicates that NP in residential areas made up 71.6% of all patches in the study area, with high-density residential areas having the most patches and low-density residential areas having the fewest patches. As regards the patch

density (PD), high-rise commercial areas had the greatest PD (133.8), followed by medium-density residential areas (PD of 110.3), and high-rise flat areas had the lowest PD (43.6). In terms of the largest patch index (LPI), low-density residential land use areas had the highest index of 9.81 and the quasi-residential class was the land use category with the smallest patch size, with a value of 0.35. A higher LPI rating for low-density residential denotes the presence of larger and better-connected green spaces in the landscape. Lower LPI values, on the other hand, imply that the green space is more fragmented, with smaller patches dispersed over the landscape. 9.81 and the quasi-residential class was the land use category with the smallest patch size, with a value of 0.35. A higher LPI rating for low-density residential denotes the presence of larger and better-connected green spaces in the landscape. Lower LPI values, on the other hand, imply that the green space is more fragmented, with smaller patches dispersed over the landscape.

In addition, the mean shape index (SHAPE_MN), which is one of the indicators of spatial configuration, showed the high complexity of the shapes of urban green spaces in all the functional land uses. The low-density residential areas had greeneries that were more complex and irregular in shape (SHAPE_MN = 1.32) than other functional land use categories. While the lowest green space shape complexities were observed in the quasi-residential areas (SHAPE_MN = 1.17).
The analysis of mean Euclidean nearest neighbour distance (ENN_MN) indicated that quasi-residential areas had the highest value of 45.54; similarly, quasi-residential and high-rise flat residential areas indicated high values among the other functional land use types in the study area. This indicates that green spaces in quasi-residential areas are more dispersed or farther apart compared to other land uses. Low clustering or concentration of green areas in the quasi-residential land use zones is indicated by the high value of ENN_MN. On the other hand, low-density residential areas had the lowest ENN_MN values of 26.53.

Table 6: Composition and configuration of UGS in functional land use in Lilongwe City

	Land Use Types	NP	PD	LPI	SHAPE_MN	ENN_MN
1	**Residential Class**					
	1.1 High Density Residential	3584	63.9	0.51	1.17	41.5
	1.2 Medium Density Residential	1976	110.3	2.30	1.25	28.3
	1.3 Low Density Residential	1387	82.5	9.81	1.32	26.5

	1.4 Quasi Residential	2779	55.3	0.35	1.17	45.5
	1.5 High Rise Flat Area	500	43.6	1.60	1.26	41.3
2	**Commercial Class**					
	2.1 Commercial Area	1301	89.3	0.71	1.20	35.0
	2.2 High Rise Commercial	287	133.8	3.99	1.25	28.54
3	**Industrial Class**					
	3.1 Heavy/Large Scale Industry	679	51.3	1.31	1.21	39.92
	3.2 Light Industry	217	50.8	2.04	1.22	35.73
4	**Government Class**	1568	90.0	4.40	1.25	31.22

Relationship between landscape metrics of urban green spaces and urban form metrics

Urban green space metrics showed some significant relationships with urban form metrics (Table 8). For instance, RD strongly negatively correlated with SHAPE_MN (-0.663*). BD strongly positively correlated with NP (0.714*), SHAPE_MN (0.681*) and ENN_MN (0.651*). This suggests that as building density increases, the number of patches (NP) in green spaces tends to increase as well. In addition, in areas with higher building density, the green spaces are farther apart from each other on average. MN_PARA had a strong negative correlation with PD (-0.785**), implying that areas with buildings that have a higher mean perimeter area ratio (indicating more irregularly shaped buildings or less open space) have lower patch density in terms of green spaces.

Table 7: Correlation between urban green space and urban form metrics

		1	2	3	4	5	6	7	8	9
1	NP	1								
2	PD	-.076	1							
3	LPI	-.230	.353	1						
4	SHAPE_MN	-.484	.335	.874**	1					
5	ENN_MN	.326	-.779**	-.744*	-.754*	1				

6	RD	.269	.189	-.445	-.663*	.276	1			
7	BD	.714*	-.336	-.424	-.681*	.651*	.471	1		
8	MN_PARA	.111	-.785**	-.260	-.207	.480	-.347	.097	1	
9	BC	.410	.113	-.335	-.557	.309	.435	.734*	-.322	1

*. Correlation is significant at the 0.05 level (2-tailed).

**. Correlation is significant at the 0.01 level (2-tailed).

Discussions

Urban green space distribution in Lilongwe City

Lilongwe City is one of the cities with abundant greenery. Despite having abundant greenery, there are disparities in the distribution of greenery among the neighbourhoods and functional land uses. The study has revealed that 20% of the neighbourhoods do not meet the minimum WHO recommended value of 9 m^2 of green space per individual [46]. This means that the available green spaces make little contribution to positive living in these neighbourhoods. It is worth noting that most of these neighbourhoods are in high-density and quasi-residential areas. These neighbourhoods contain about 50% of the total population of the city. This shows how serious the disparities are in the distribution of UGS in the city.

In addition, the study revealed that population density was negatively correlated with urban green space. Numerous factors, like a shortage of adequate land in densely populated neighbourhoods or encroachment on areas allocated for greenery, may contribute to this [27]. The quasi-residential areas are the informal settlements, and some of these areas have encroached on environmentally sensitive areas with little space to provide them with green spaces. These results are consistent with those of Bille et al. [11], who investigated how population density affects patterns in urban green space around the world. They found a rapid decline in UGS coverage in areas with high population densities. Other authors have also observed the opposite effect of population on urban green spaces [47,48].

In Lilongwe City, most of the people who live in these undersupplied areas (quasi and high-density residential neighbourhoods) are low-incoming residents. In Lilongwe City, 73% of the residential land share is made up of informal settlements and low-income neighbourhoods [49]. Disparities in green space distributions were also found in Southeast Asia [50], South Africa [19], Guangzhou,

China [20], and Brisbane, Adelaide, Sydney, Perth, and Melbourne [51], with low availability of greeneries found in low-income neighbourhoods. Similarly, the poorest neighbourhoods were observed to have limited access to green and blue spaces in Hong Kong [52].

Urban dwellers who are deprived of the many advantages of UGS may be concerned about uneven distribution and a lack of UGS. The results highlight the difficulties and need for sustainable urban planning and development to ensure equity in the distribution and accessibility of UGS to all city dwellers. Lack of access to green spaces in densely populated areas can exacerbate already-existing health and environmental injustice disparities.

Urban green composition and configuration

As regards to composition of UGS in functional land uses, the study has revealed that about 59.7% of UGS in Lilongwe City is found in functional land uses. Out of this, 67.6% of UGS were found in residential zones. A high proportion of UGS in the residential areas was due to their relatively large areas. In functional land uses, residential usage accounts for around 77% of the total area.

In addition, the mean shape index (SHAPE_MN), which is one of the indicators of spatial configuration, showed the high complexity of the shapes of urban green spaces in all the functional land uses. The low-density residential areas had greeneries that were more complex and irregular in shape than other functional land use categories. While the lowest green space shape complexities were observed in the quasi-residential areas. The complexity of green spaces in low-density residential areas may be attributed to the abundance of greenery in these areas, hence providing a variety of shapes. Studies have shown that more complex and irregularly shaped green spaces may provide higher aesthetic, recreational, ecological and health benefits since they interact well with the surrounding area [53–55]. This implies that people who live in the low-density residential areas are more advantaged than those in the informal/quasi-residential areas in Lilongwe City.

Additionally, the study revealed that across all functional land uses, the largest patch index values were comparatively low. Most of the functional land uses have an LPI of less than 3.0. Again, densely populated areas showed the lowest LPI levels, which may be attributed to more structures

and people habits in these places leading to fragmentation. The low LPI number also highlights the possibility of disparities in how different land use groups have access to green spaces.

The analysis of the mean Euclidean nearest neighbour distance (ENN_MN) indicated that quasi-residential areas had the highest value of 45.54; similarly, quasi-residential and high-rise flat residential areas are more dispersed or farther apart compared to other land uses. The high value of ENN_MN indicates that green areas are sparsely clustered or concentrated within the quasi-residential land use zones. On the other hand, low-density residential areas had the lowest ENN_MN values.

The composition and configuration of urban green spaces in functional land uses highlight the challenges and need for sustainable urban planning and development to ensure equity in the distribution and accessibility of UGS to all city dwellers. Urban dwellers who are deprived of the many advantages of UGS may be concerned about uneven distribution and a lack of UGS. The lack of access to green spaces in densely populated areas can exacerbate already-existing health and environmental injustice disparities.

The association between urban green space and urban form metrics and implications for urban planning

The study revealed significant correlations between urban green space and urban form. For instance, BD is strongly positively correlated with NP, SHAPE_MN, and ENN_MN. This suggests that as building density increases, the number of patches (NP) of green spaces tends to increase as well. In addition, in areas with higher building density, the green spaces are farther apart from each other, with their shapes becoming more complex. The severity of fragmentation increases with the number of green space patches [56]. High dispersion and isolation of UGS patches may be associated with the increase in building density. This agrees with the finding by Yeh et al. [57], who found that the distances between green spaces in highly urbanised areas were higher than those in the less urbanised areas in Taipei Metropolitan, Taiwan.

BC strongly positively correlated with LPI. This implies that as the areas within the city become more urbanised and built-up (increased building coverage), larger and more connected green spaces may be present. Similarly, the building perimeter area ratio (MN_PARA), which is a measure of the complexity of the built-up area, showed a significant negative relationship with PD of green space. We found that areas with compact building forms were less fragmented. This could be due to urban planning interventions that prioritise larger green areas within the central areas of the city [27]. Lilongwe City was built on the garden city concept and has abundant large patches of green space, such as nature sanctuaries, botanic gardens, and golf courses within the central part of the city [27]. Contrary to the findings by Huang et al. [44] and Bereitschaft & Debbage [38], who found that cities with complex urban areas (higher PARA value) had high fragmentation of UGS, Compact building forms like high-rise buildings and mixed-use developments may improve UGS efficiency since they use land more effectively and free up more space for green spaces [58]. This is accomplished through checking urban sprawl and maximising land usage, which increases the overall green space per capita in denser neighbourhoods. However, compact building forms may result in higher population densities, which may lead to the loss of existing greenery to pave the way for buildings and infrastructure to accommodate the huge populations. Compact building forms can cause UGS fragmentation.

The relationship between the distribution and availability of UGS and urban form and design can be two-way. Urban green space is significantly correlated with urban form, which suggests that urban form is crucial to understanding the distribution and configuration of UGS. Urban development plans should incorporate green spaces and consider their significance. Smart Growth Strategies that prioritize compact and mixed-use development can help with this [58,59]. These strategies try to preserve or improve green spaces while increasing road density, building density and coverage. To achieve liveability and sustainability of neighbourhoods, the city should take a balance that integrates green spaces into the urban fabric. The purpose of the present research was to establish relationships between UGS and urban form. To better understand the nature of the association as well as account for confounding factors, future research should consider establishing a cause-and-effect relationship between urban form and UGS.

Conclusions

The study has revealed that urban green spaces (UGS) are distributed unevenly among different neighbourhoods and functional land uses in Lilongwe City, despite the city's abundance of green spaces. A sizeable fraction of the populace resides in places with insufficient access to the recommended amounts of green space per person. The study has also revealed that population density is one of the factors that is influencing the availability and distribution of UGS as low green space coverage was observed in highly populated neighbourhoods. A low supply of UGS in low-income, high-density and informal settlements may raise concerns about environmental justice. Therefore, deliberate efforts should be undertaken to establish green areas in neighbourhoods with lower urban green space coverage to enhance the liveability and inequities in the distribution of UGS in Lilongwe City. The study has also shown that the spatial composition and configuration of UGS in functional is complex and differed with the land use class. However, low-density and quasi-residential land uses were found to have more intricate green spaces. It was also revealed that urban form had an impact on the composition and configuration of UGS. Urban form indicators like building coverage, building density, and road density had significant associations with the distribution and configuration of urban green spaces. Urban form and green space have a complicated and multifaceted interaction. Therefore, urban planning and designs that consider the built environment as well as green spaces as essential elements are necessary to achieve sustainability and liveability in the city. To ensure that there is equitable distribution and accessibility, green spaces should be integrated into the urban fabric. This can be achieved by promoting urban smart growth strategies and policies, which can help strike a balance between development and the preservation of open space while promoting compact and mixed-use development.

References

1. Tonne C, Adair L, Adlakha D, Anguelovski I, Belesova K, Berger M, et al. Defining pathways to healthy sustainable urban development. Environ Int. 2021;146: 106236. doi:10.1016/j.envint.2020.106236

2. Sinha BRK. Multidimensional Approach to Quality of Life Issues, A Spatial Analysis. 2019; 3–23. doi:10.1007/978-981-13-6958-2_1

3. Khoshkar S, Balfors B, Wärnbäck A. Planning for green qualities in the densification of suburban Stockholm – opportunities and challenges. J Environ Plan Manag. 2018;61: 2613–2635. doi:10.1080/09640568.2017.1406342

4. Sikorska D, Wojnowska-Heciak M, Heciak J, Bukowska J, Łaszkiewicz E, Hopkins RJ, et al. Rethinking Urban Green Spaces for urban resilience. Do green spaces need adaptation to meet public post-covid expectations? Urban For Urban Green. 2023;80: 127838. doi:10.1016/j.ufug.2023.127838

5. Junior DPM, Bueno C, Silva CM da. The Effect of Urban Green Spaces on Reduction of Particulate Matter Concentration. Bull Environ Contam Toxicol. 2022;108: 1104–1110. doi:10.1007/s00128-022-03460-3

6. Terkenli TS, Bell S, Živojinović I, Tomićević-Dubljević J, Panagopoulos T, Straupe I, et al. The Urban Forest, Cultivating Green Infrastructure for People and the Environment. Futur City. 2017; 191–216. doi:10.1007/978-3-319-50280-9_16

7. Semeraro T, Scarano A, Buccolieri R, Santino A, Aarrevaara E. Planning of Urban Green Spaces: An Ecological Perspective on Human Benefits. Land. 2021;10: 105. doi:10.3390/land10020105

8. Lindemann-Matthies P, Brieger H. Does urban gardening increase aesthetic quality of urban areas? A case study from Germany. Urban For Urban Green. 2016;17: 33–41. doi:10.1016/j.ufug.2016.03.010

9. Chang L-S, Bisgrove RJ, Liao M-Y. Improving educational functions in botanic gardens by employing landscape narratives. Landsc Urban Plan. 2008;86: 233–247. doi:10.1016/j.landurbplan.2008.03.003

10. Wortzel JD, Wiebe DJ, DiDomenico GE, Visoki E, South E, Tam V, et al. Association Between Urban Greenspace and Mental Wellbeing During the COVID-19 Pandemic in a U.S. Cohort. Front Sustain Cities. 2021;3: 686159. doi:10.3389/frsc.2021.686159

11. Bille RA, Jensen KE, Buitenwerf R. Global patterns in urban green space are strongly linked to human development and population density. Urban For Urban Green. 2023;86: 127980. doi:10.1016/j.ufug.2023.127980

12. Chen J, Kinoshita T, Li H, Luo S, Su D, Yang X, et al. Toward green equity: An extensive study on urban form and green space equity for shrinking cities. Sustain Cities Soc. 2023;90: 104395. doi:10.1016/j.scs.2023.104395

13. Colding J, Lundberg J, Folke C. Incorporating Green-area User Groups in Urban Ecosystem Management. AMBIO: A J Hum Environ. 2006;35: 237–244. doi:10.1579/05-a-098r.1

14. Bush J. The role of local government greening policies in the transition towards nature-based cities. Environ Innov Soc Transit. 2020;35: 35–44. doi:10.1016/j.eist.2020.01.015

15. Garcia-Garcia MJ, Christien L, García-Escalona E, González-García C. Sensitivity of green spaces to the process of urban planning. Three case studies of Madrid (Spain). Cities. 2020;100: 102655. doi:10.1016/j.cities.2020.102655

16. Gulsrud NM, Hertzog K, Shears I. Innovative urban forestry governance in Melbourne?: Investigating "green placemaking" as a nature-based solution. Environ Res. 2018;161: 158–167. doi:10.1016/j.envres.2017.11.005

17. Csomós G, Farkas JZ, Kovács Z. Access to urban green spaces and environmental inequality in post-socialist cities. Hung Geogr Bull. 2020;69: 191–207. doi:10.15201/hungeobull.69.2.7

18. Richards DR, Passy P, Oh RRY. Impacts of population density and wealth on the quantity and structure of urban green space in tropical Southeast Asia. Landsc Urban Plan. 2017;157: 553–560. doi:10.1016/j.landurbplan.2016.09.005

19. Venter ZS, Shackleton CM, Staden FV, Selomane O, Masterson VA. Green Apartheid: Urban green infrastructure remains unequally distributed across income and race geographies in South Africa. Landsc Urban Plan. 2020;203: 103889. doi:10.1016/j.landurbplan.2020.103889

20. Yang W, Yang R, Zhou S. The spatial heterogeneity of urban green space inequity from a perspective of the vulnerable: A case study of Guangzhou, China. Cities. 2022;130: 103855. doi:10.1016/j.cities.2022.103855

21. Mawenda J, Watanabe T, Avtar R. An Analysis of Urban Land Use/Land Cover Changes in Blantyre City, Southern Malawi (1994–2018). Sustainability. 2020;12: 2377. doi:10.3390/su12062377

22. Mungai LM, Messina JP, Zulu LC, Qi J, Snapp S. Modeling Spatiotemporal Patterns of Land Use/Land Cover Change in Central Malawi Using a Neural Network Model. Remote Sens. 2022;14: 3477. doi:10.3390/rs14143477

23. Munthali MG, Davis N, Adeola AM, Botai JO, Kamwi JM, Chisale HLW, et al. Local Perception of Drivers of Land-Use and Land-Cover Change Dynamics across Dedza District, Central Malawi Region. Sustainability. 2019;11: 832. doi:10.3390/su11030832

24. Nazombe K, Nambazo O. Monitoring and assessment of urban green space loss and fragmentation using remote sensing data in the four cities of Malawi from 1986 to 2021. Sci Afr. 2023;20: e01639. doi:10.1016/j.sciaf.2023.e01639

25. Chen Y, Jones CA, Dunse NA, Li E, Liu Y. Housing Prices and the Characteristics of Nearby Green Space: Does Landscape Pattern Index Matter? Evidence from Metropolitan Area. Land. 2023;12: 496. doi:10.3390/land12020496

26. NSO. 2018 Malawi Population and Housing Census Report. 2023 Jul. Available: https://malawi.unfpa.org/sites/default/files/resource-

pdf/2018%20Malawi%20Population%20and%20Housing%20Census%20Main%20Report%20%281%29.pdf

27. JICA. The Study on Urban Development Master Plan for Lilongwe in the Republic of Malawi. KRI International Corp; 2010 Nov. Available: https://openjicareport.jica.go.jp/pdf/12003752_01.pdf

28. MalawiGovernment. MALAWI GOVERNMENT NATIONAL URBAN POLICY APRIL 2019. Jul 11, 2023. Available: file:///C:/Users/Nazommbe/Downloads/2019%20April%20National%20Urban%20Policy_Signed.pdf

29. Li Q, Peng K, Cheng P. Community-Level Urban Green Space Equity Evaluation Based on Spatial Design Network Analysis (sDNA): A Case Study of Central Wuhan, China. Int J Environ Res Public Heal. 2021;18: 10174. doi:10.3390/ijerph181910174

30. Davies RG, Barbosa O, Fuller RA, Tratalos J, Burke N, Lewis D, et al. City-wide relationships between green spaces, urban land use and topography. Urban Ecosyst. 2008;11: 269. doi:10.1007/s11252-008-0062-y

31. Park J-H, Cho G-H. Examining the Association between Physical Characteristics of Green Space and Land Surface Temperature: A Case Study of Ulsan, Korea. Sustainability. 2016;8: 777. doi:10.3390/su8080777

32. Robinson JM, Mavoa S, Robinson K, Brindley P. Urban centre green metrics in Great Britain: A geospatial and socioecological study. PLOS ONE. 2022;17: e0276962. doi: 10.1371/journal.pone.0276962

33. Shekhar S, Aryal J. Role of geospatial technology in understanding urban green space of Kalaburagi city for sustainable planning. Urban For Urban Green. 2019;46: 126450. doi: 10.1016/j.ufug.2019.126450

34. Viana CM, Oliveira S, Oliveira SC, Rocha J. Spatial Modeling in GIS and R for Earth and Environmental Sciences. 2019; 621–651. doi:10.1016/b978-0-12-815226-3.00029-6

35. Chung S, Breshears LE, Yoon J-Y. Smartphone near infrared monitoring of plant stress. Comput Electron Agric. 2018;154: 93–98. doi:10.1016/j.compag.2018.08.046

36. You H. Characterizing the inequalities in urban public green space provision in Shenzhen, China. Habitat Int. 2016;56: 176–180. doi:10.1016/j.habitatint.2016.05.006

37. Nero BF. Urban green space dynamics and socio-environmental inequity: multi-resolution and spatiotemporal data analysis of Kumasi, Ghana. Int J Remote Sens. 2017;38: 6993–7020. doi:10.1080/01431161.2017.1370152

38. Bereitschaft B, Debbage K. Regional Variations in Urban Fragmentation Among U.S. Metropolitan and Megapolitan Areas. Appl Spat Anal Polic. 2014;7: 119–147. doi:10.1007/s12061-013-9092-9

39. Guan Y, Li X, Li S, Sun H, Liu H. Effect of Urban fringes green space fragmentation on ecosystem service value. PLoS ONE. 2022;17: e0263452. doi:10.1371/journal.pone.0263452

40. Woldesemayat EM, Genovese PV. Urban Green Space Composition and Configuration in Functional Land Use Areas in Addis Ababa, Ethiopia, and Their Relationship with Urban Form. Land. 2021;10: 85. doi:10.3390/land10010085

41. Rezaei N, Millard-Ball A. Urban form and its impacts on air pollution and access to green space: A global analysis of 462 cities. PLOS ONE. 2023;18: e0278265. doi:10.1371/journal.pone.0278265

42. Margaritis E, Kang J. Relationship between green space-related morphology and noise pollution. Ecol Indic. 2017;72: 921–933. doi:10.1016/j.ecolind.2016.09.032

43. Tratalos J, Fuller RA, Warren PH, Davies RG, Gaston KJ. Urban form, biodiversity potential and ecosystem services. Landsc Urban Plan. 2007;83: 308–317. doi:10.1016/j.landurbplan.2007.05.003

44. Huang C, Yang J, Jiang P. Assessing Impacts of Urban Form on Landscape Structure of Urban Green Spaces in China Using Landsat Images Based on Google Earth Engine. Remote Sens. 2018;10: 1569. doi:10.3390/rs10101569

45. Faizi N, Alvi Y. Biostatistics Manual for Health Research. 2023; 109–126. doi:10.1016/b978-0-443-18550-2.00002-5

46. Russo A, Cirella GT. Modern Compact Cities: How Much Greenery Do We Need? Int J Environ Res Public Heal. 2018;15: 2180. doi:10.3390/ijerph15102180

47. Li F, Wang X, Liu H, Li X, Zhang X, Sun Y, et al. Does economic development improve urban greening? Evidence from 289 cities in China using spatial regression models. Environ Monit Assess. 2018;190: 541. doi:10.1007/s10661-018-6871-4

48. Zhang W, Randall M, Jensen MB, Brandt M, Wang Q, Fensholt R. Socio-economic and climatic changes lead to contrasting global urban vegetation trends. Glob Environ Chang. 2021;71: 102385. doi:10.1016/j.gloenvcha.2021.102385

49. UN-Habitat. Malawi: Lilongwe urban profile. 8 Nov 2011 [cited 2 Nov 2023]. Available: https://www.zaragoza.es/contenidos/medioambiente/onu/issue06/1136-eng.pdf

50. Vaz AS, Kueffer C, Kull CA, Richardson DM, Vicente JR, Kühn I, et al. Integrating ecosystem services and disservices: insights from plant invasions. Ecosyst Serv. 2017;23: 94–107. doi:10.1016/j.ecoser.2016.11.017

51. Astell-Burt T, Feng X, Mavoa S, Badland HM, Giles-Corti B. Do low-income neighbourhoods have the least green space? A cross-sectional study of Australia's most populous cities. BMC Public Heal. 2014;14: 292. doi:10.1186/1471-2458-14-292

52. Liu D, Kwan M-P. Integrated analysis of doubly disadvantaged neighborhoods by considering both green space and blue space accessibility and COVID-19 infection risk. PLOS ONE. 2022;17: e0273125. doi:10.1371/journal.pone.0273125

53. Alcock I, White MP, Wheeler BW, Fleming LE, Depledge MH. Longitudinal Effects on Mental Health of Moving to Greener and Less Green Urban Areas. Environ Sci Technol. 2014;48: 1247–1255. doi:10.1021/es403688w

54. Sodoudi S, Zhang H, Chi X, Müller F, Li H. The influence of spatial configuration of green areas on microclimate and thermal comfort. Urban For Urban Green. 2018;34: 85–96. doi:10.1016/j.ufug.2018.06.002

55. Vos CC, Berry P, Opdam P, Baveco H, Nijhof B, O'Hanley J, et al. Adapting landscapes to climate change: examples of climate- proof ecosystem networks and priority adaptation zones. J Appl Ecol. 2008;45: 1722–1731. doi:10.1111/j.1365-2664.2008.01569.x

56. Rahimi E, Barghjelveh S, Dong P. Using the Lonsdorf model for estimating habitat loss and fragmentation effects on pollination service. Ecol Process. 2021;10: 22. doi:10.1186/s13717-021-00291-8

57. Yeh C-T, Cheng Y-Y, Liu T-Y. Spatial Characteristics of Urban Green Spaces and Human Health: An Exploratory Analysis of Canonical Correlation. Int J Environ Res Public Heal. 2020;17: 3227. doi:10.3390/ijerph17093227

58. Fan P, Lee Y-C, Ouyang Z, Huang S-L. Compact and green urban development—towards a framework to assess urban development for a high-density metropolis. Environ Res Lett. 2019;14: 115006. doi:10.1088/1748-9326/ab4635

59. Balikçi S, Giezen M, Arundel R. The paradox of planning the compact and green city: analyzing land-use change in Amsterdam and Brussels. J Environ Plan Manag. 2022;65: 2387–2411. doi:10.1080/09640568.2021.1971069

Figure 3 Click here to access/download;Figure;Fig 3.tif

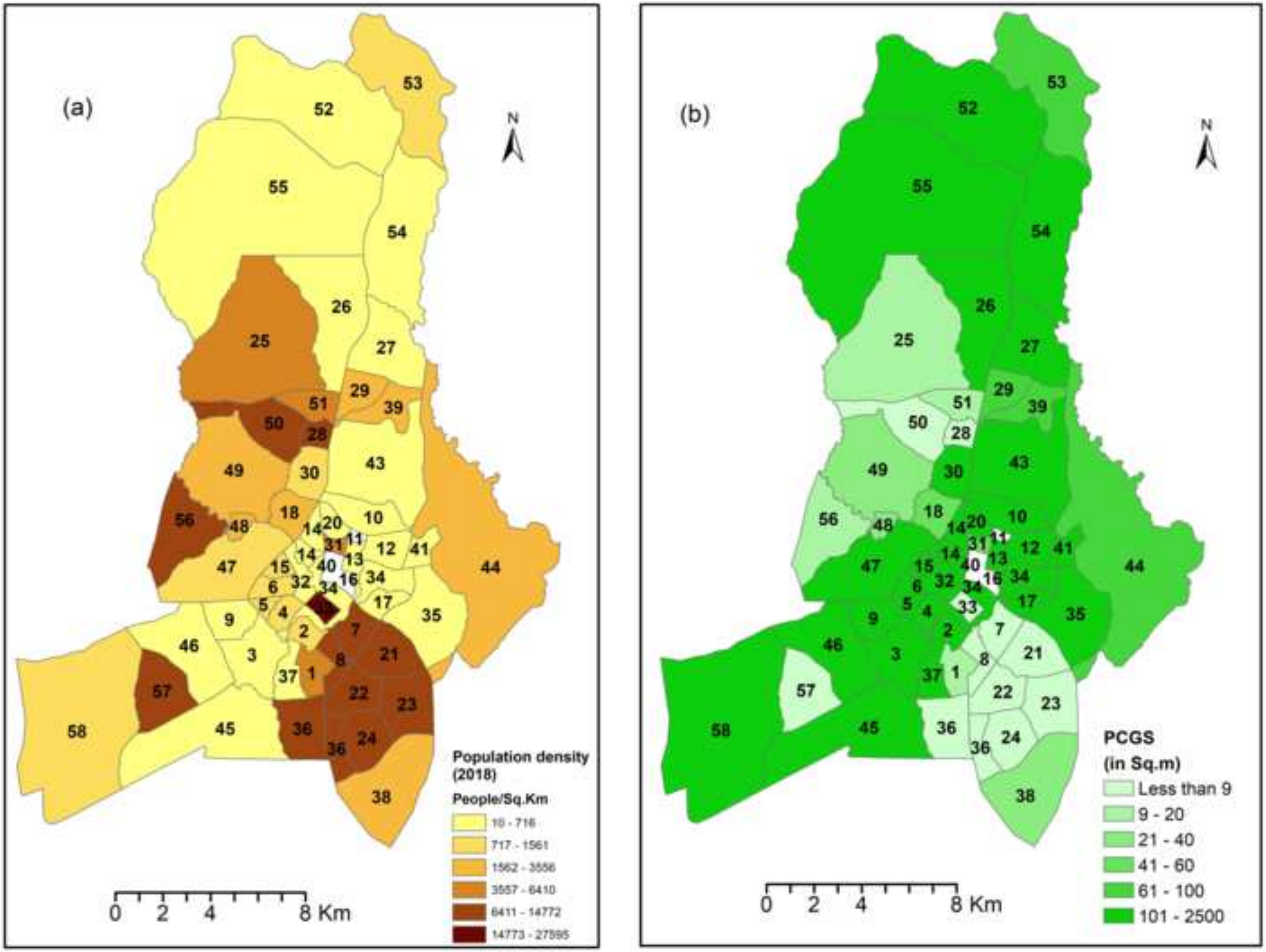

Figure 1 Click here to access/download;Figure;Fig 1.tif

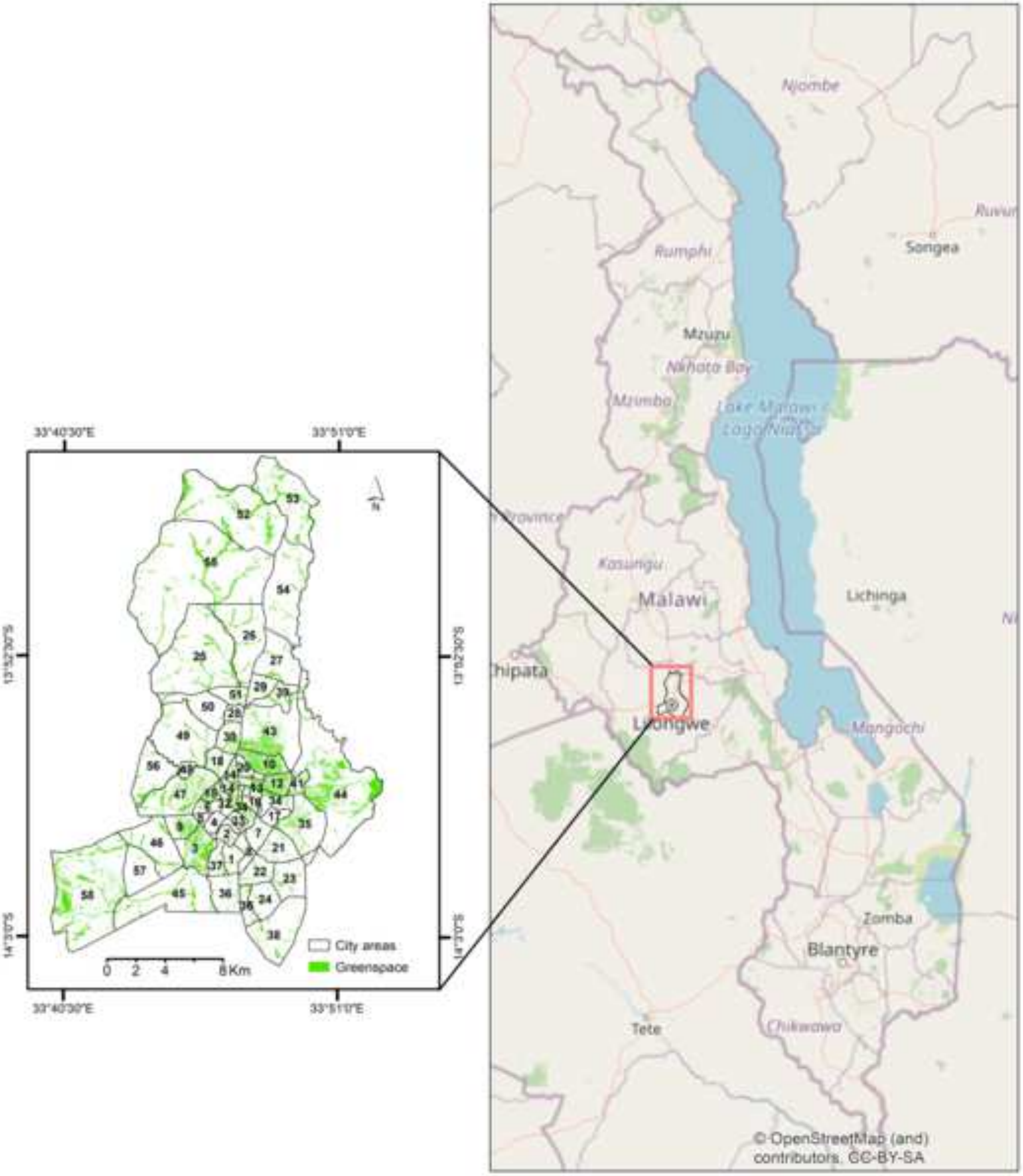

Figure 2

Click here to access/download;Figure;Fig 2.tif

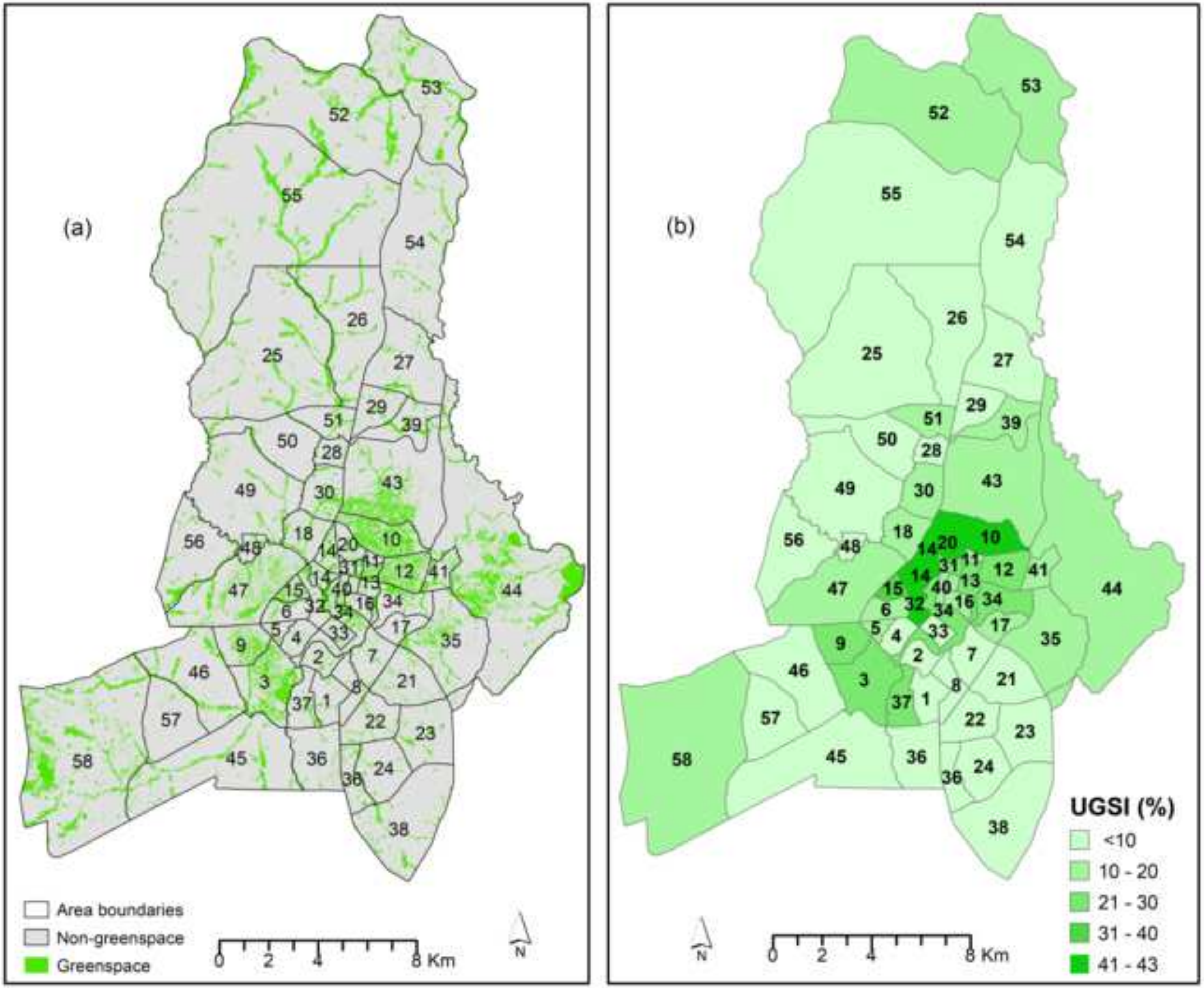

Author contributions

Click here to access/download

Supporting Information

Author_contributions.docx

Competing interest statement

Click here to access/download
Supporting Information
Competing interests.docx

Click here to access/download
Supporting Information
Financial Disclosure Statement.docx
