# Supplementary material for: The spatial heterogeneity of urban green space distribution and configuration in Lilongwe City, Malawi
Source: PLoS One. 2024 Jul 24;19(7):e0307518. doi: 10.1371/journal.pone.0307518 (PMC11268670; doi:10.1371/journal.pone.0307518)
Supplement: Attachment — Submitted filename: Response to Reviewers.docx [file pone.0307518.s002.docx]

**Responses to reviewer’s comments**

*Reviewer #1: The added tables and especially the prepared diagram helped me understand the topic better. However, figures 2 and 3 would have been better if they had not been deleted because they better showed the population distribution and green space.*

Response: We have reinstated figures 2 and 3, now figures 3 and 4 respectively. These figures have been prepared by the authors. Figure 3 is derived from sentinel 2 images. These images have no restrictions on reuse, sale, or redistribution. They only require that the author include a statement of the data source in their manuscripts. Figure 4 was prepared from the public report, the Malawi population and housing census 2018.

*Reviewer #2: The manuscript describe a technically sound piece of scientific research with data that supports the conclusions. All comments have been addressed in the revised manuscript.*

Response: Thank you
